# Mechanisms of action of hESC-secreted proteins that enhance human and mouse myogenesis

**DOI:** 10.18632/aging.100659

**Published:** 2014-05-11

**Authors:** Hanadie Yousef, Michael J. Conboy, Hikaru Mamiya, Matthew Zeiderman, Christina Schlesinger, David V. Schaffer, Irina M. Conboy

**Affiliations:** ^1^Department of Bioengineering and California Institute for Quantitative Biosciences (QB3), UC Berkeley, Berkeley, CA 94720, USA; ^2^Department of Molecular and Cellular Biology, UC Berkeley, Berkeley, CA 94720, USA; ^3^Department of Chemical and Biomolecular Engineering and Helen Wills Neuroscience Institute, UC Berkeley, Berkeley, CA 94720 USA

**Keywords:** hESC, muscle, myogenesis, Notch, MAPK, aging, rejuvenation, stem cell

## Abstract

Adult stem cells grow poorly in vitro compared to embryonic stem cells, and in vivo stem cell maintenance and proliferation by tissue niches progressively deteriorates with age. We previously reported that factors produced by human embryonic stem cells (hESCs) support a robust regenerative capacity for adult and old mouse muscle stem/progenitor cells. Here we extend these findings to human muscle progenitors and investigate underlying molecular mechanisms. Our results demonstrate that hESC-conditioned medium enhanced the proliferation of mouse and human muscle progenitors. Furthermore, hESC-produced factors activated MAPK and Notch signaling in human myogenic progenitors, and Delta/Notch-1 activation was dependent on MAPK/pERK. The Wnt, TGF-β and BMP/pSmad1,5,8 pathways were unresponsive to hESC-produced factors, but BMP signaling was dependent on intact MAPK/pERK. c-Myc, p57, and p18 were key effectors of the enhanced myogenesis promoted by the hECS factors. To define some of the active ingredients of the hESC-secretome which may have therapeutic potential, a comparative proteomic antibody array analysis was performed and identified several putative proteins, including FGF2, 6 and 19 which as ligands for MAPK signaling, were investigated in more detail. These studies emphasize that a “youthful” signaling of multiple signaling pathways is responsible for the pro-regenerative activity of the hESC factors.

## INTRODUCTION

Adult stem cells persist in the body as we age, but their regenerative capacity declines over time, leading to an inability of tissues and organs to maintain homeostasis and repair damage with advancing age. Old skeletal muscle loses its regenerative ability due to the failure of satellite cells (muscle stem cells) to divide and generate fusion competent myoblasts and terminally differentiated myofibers in response to muscle injury or attrition [1,2]. Consequentially, the replacement of the damaged tissue with new muscle fibers becomes in-efficient with age, and instead scarring and inflammation persist [3,4]. This age-specific decline in myogenic proliferation results from changes in key cellular signaling that, in turn, is affected by molecular changes in the stem cell niches. Among many other changes, decreased Notch and mitogen-activated protein kinase (MAPK) pathway signaling, as well as mis-regulation of the transforming growth factor-β (TGF-β), tumor necrosis factor-α (TNF-α), and Wnt pathways, have each been shown to underlie the age-specific decline in muscle regeneration [1,2].

Notch activation is a key age-specific determinant of satellite cell and myoblast proliferation, and the expression of constitutively active Notch-1 enhances the proliferation of a variety of myogenic cells and delays their differentiation into myofibers during normal adult myogenesis by antagonism with Wnt [3-5]. The level of Notch-1 activation in response to injury decreases with age in satellite cells due to the failure of old damaged muscle fibers to up-regulate the Notch-1 ligand Delta and forced inhibition of Notch-1 in young muscle perturbs the activation of young satellite cells and interferes with productive repair of young tissue [6]. In agreement with this role of Notch, a conditional knock-out of RBP-Jκ (one of the main activating / repressing target loci of Notch-1-3 signaling [7], diminishes the ability of satellite cells to self-renew and promotes their precocious differentiation [8, 9]. Interestingly, it has also been shown that the crosstalk between the MAPK and Notch pathways is essential and evolutionarily conserved in the invertebrate embryonic development of *C. elegans* and D. *melanogaster*, where the MAPK pathway positively regulates Delta expression and thus subsequent activation of Notch, in a variety of cell-fate specification processes [10-12]. Furthermore, some evidence also exists for cross-talk between Notch and MAPK in developing and postnatal mammalian tissues [2, 10-12]. In muscle, once the blockage in the activation of aged satellite cells is overcome (for example by activation of Notch or by culture in mitogenic growth medium), their abilities to form myotubes are as robust as those of young cells both in vitro and in vivo [3, 6, 13, 14].

Finally, the bone morphogenetic protein, BMP, signaling pathway has been shown to be a critical regulator of various embryonic and adult stem cell niches [14,15]. BMP is a ligand for the TGF-β protein superfamily and its signal transduction operates through the Smad1, 5 and 8 transcription factors, which become phosphorylated and activated by BMP receptors and form heterodimers with constitutively present Smad4. Such events promote nuclear translocation of these transcriptional regulators causing changes in expression of hundreds of down-stream target genes. In addition to the canonical Smad signaling, the MAPK pathway can also be induced by BMP [15]. In adult myogenesis, BMP signaling is upregulated after satellite cell activation both in vivo and in vitro, and inhibition of BMP signaling promotes myogenic differentiation [16,17]. BMPs may promote satellite cell proliferation by activating their downstream targets, the differentiation-inhibiting Id genes, which inhibit transcription factors that promote cell differentiation [17].

Previous studies have demonstrated that aging of the stem cell niche is responsible for the decline of tissue regeneration and productive homeostasis not only in skeletal muscle but also in a variety of postnatal tissues, and that old muscle can be rejuvenated to repair almost as well as young through several means [4]. These findings may prove to be important for the development of therapies for age-related tissue degeneration and trauma. However, not all of the factors that influence the niche are known, and the various physiological molecules and balance of signaling crosstalk that modulate healthy regeneration are not well established. In addition, while numerous approaches have been utilized to reverse age-related tissue deterioration in murine models, none are suitable for clinical translation. As one example, skewing the signaling strength of one pathway (either up or down) over a long timespan is likely to be deleterious for cells and tissues, potentially leading to more cellular dysregulation or oncogenic progression [18]. In contrast, modulation of multiple interactive signaling pathways to their “youthful” levels may have beneficial effects on tissue repair and maintenance.

We previously established that hESC-produced proteins enhance the regenerative capacity of postnatal and old mouse muscle stem/progenitor cells, and that MAPK is indispensable for these pro-regenerative effects [14]. Furthermore, many FGFs bind heparin [16], and we demonstrated that the heparin-binding fraction of the hESC-produced factors exert robust proliferative effects on mouse muscle progenitor cells and provide a rejuvenating stimulus for old murine muscle regeneration [17]. Here, we show that hESC-produced proteins enhance the regenerative capacity not only of mouse but also human muscle progenitor cells, determine the molecular mechanisms by which hESC-produced proteins enhance human myogenic proliferation, and establish the molecular identity of several of these clinically relevant factors.

## RESULTS

### hESC-produced proteins enhance both mouse and human myoblast proliferation

**Figure 1:**
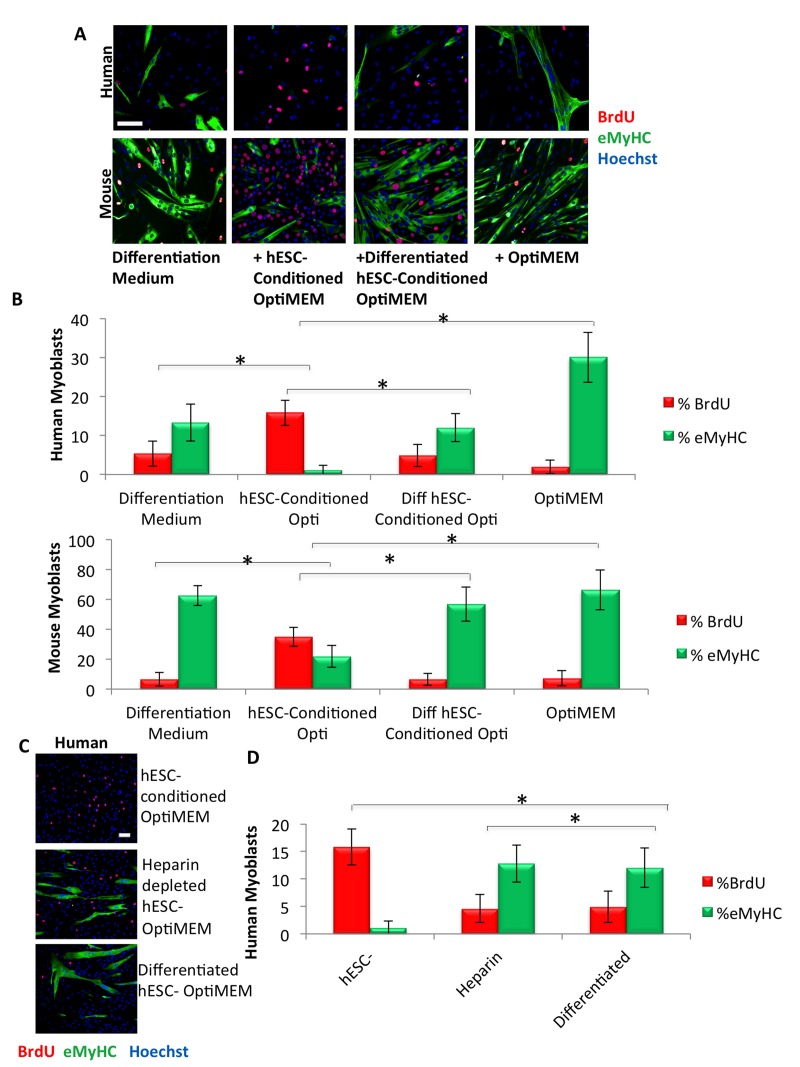
Pro-regenerative embryonic factors that enhance human and mouse myoblast proliferation contain heparin binding domains (**A**) Primary human or mouse myoblasts were cultured for 24 hours or 72 hours, respectively, in 50% differentiation medium (DMEM, 2% horse serum) plus 50% of the specified medium, with daily medium changes. A 4 hour or 2 hour BrdU pulse on human or mouse myoblasts, respectively, was performed before cell fixation to label proliferating cells. Immunofluorescence was performed for eMyHC (green) and BrdU (red), with Hoechst (blue) labeling all nuclei. Representative images are shown. Scale bar = 100 μM (**B**) Proliferation and differentiation of fusion-competent myoblasts were quantified by cell scoring in 25-50 random fields of each condition using a Molecular Devices MetaXpress automated imager and cell scoring software. Results are displayed as the mean percent of BrdU+ or eMyHC+ proliferating or differentiating cells +/−SD, respectively (n=6). Significant differences were identified by Student's t-tests (*p<0.004 for human cells; *p<8×10^−19^ for mouse cells). (**C**) Primary human myoblasts were cultured, BrdU pulsed, immunostained and quantified as in (**A**), with the specified medium. Representative images are shown. Scale bar = 100 μM (**D**) Proliferation and differentiation of fusion-competent human myoblasts were quantified as in (**B**). Results are displayed as the mean percent of BrdU+ or eMyHC+ proliferating or differentiating cells +/−SD, respectively (n=6). Significant differences were identified by Student's t-tests (*p<0.004).

To test the hypothesis that hESC-conditioned medium promotes the proliferation and inhibits the differentiation of both mouse and human muscle progenitor cells, we collected hESC-conditioned medium by incubating hESCs overnight in a serum and growth factor free medium (Opti-MEM). Primary human and mouse myoblasts (muscle progenitor cells) were cultured in a 50/50 mixture of low-mitogen “differentiation medium” (DMEM with 2% horse serum) and hESC-conditioned Opti-MEM, either for 72 hours with daily medium changes (human myoblasts) or overnight (mouse myoblasts). We used two assays of myogenic regenerative activity: the proliferation of human and mouse myoblasts by incorporation of BrdU, and their resistance to differentiation in this otherwise low-mitogen differentiation medium, as measured by expression of embryonic myosin heavy chain (eMyHC). Proliferation was significantly enhanced and differentiation inhibited in both human and mouse muscle progenitor cells by the hESC-conditioned medium (Figure 1A, B). Analogously, hESC growth medium (mTeSR1) conditioned by hESC culture enhanced myoblast proliferation more effectively than mTeSR1 alone, though mouse myoblasts exhibited significantly enhanced proliferation in both media (Supplementary Figure 1A, B and [17]).

We recently published that in the mouse system, the pro-myogenic activity of hESC-conditioned Opti-MEM is contained in proteins with heparin-binding activity [17]. To explore the evolutionary conservation of this phenomenon in a human system, we cultured primary human muscle progenitor cells in a 50/50 mixture of the low-mitogen differentiation medium plus hESC-conditioned Opti-MEM that was either complete or depleted of heparin-binding proteins. As shown in Figure 1 (C, D), the proliferation of human myogenic cells is greatly enhanced and their differentiation is inhibited by the hESC-conditioned Opti-MEM, but this pro-myogenic effect is lost when heparin-bound proteins are depleted.

To further assess the pro-regenerative activity of hESC-conditioned medium, we assayed the myogenic properties of muscle regenerative cells derived from old (2 year) mice, using our published methods [18,20]. Gastrocnemius muscles were injured using cardiotoxin (CTX), and muscle tissue was collected three days post injury. Activated satellite cells associated with muscle fibers were plated overnight in a 50/50 mixture of Opti-MEM and hESC-conditioned Opti-MEM, with 5% old serum. In agreement with previously published work [14], exposure to hESC-conditioned Opti-MEM more than doubled the percentage of BrdU+/Desmin+ old activated satellite cells isolated from regenerating muscle, bringing them close to the percentage of young activated satellite cells (Supplementary Figure 2A, B). These data demonstrate that the hESC-conditioned Opti-MEM exerts a strong pro-myogenic activity on not only mouse but also human muscle progenitor cells, as well as positively influences the myogenicity of freshly-isolated muscle cells that respond to tissue injury, even in an old environment or when cultured in old serum where their myogenicity is typically suppressed.

### Identifying the molecular mechanisms by which hESC-produced factors enhance the regenerative responses of human and mouse muscle progenitor cells

**Figure 2:**
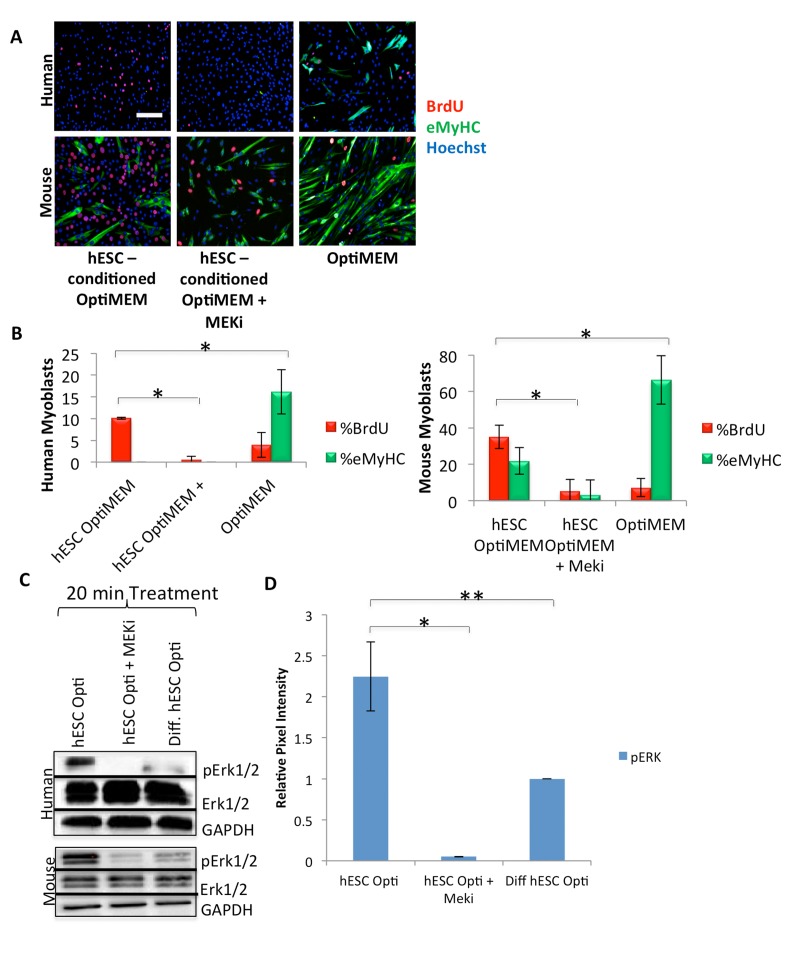
hESCs secrete pro-myogenic proteins that act primarily through MAPK signaling (**A**) Primary human or mouse myoblasts were cultured and BrdU pulsed as in Figure 1, with or without 10 μM of a MAPK pathway inhibitor (MEKi), with daily medium changes, and immunostained as in Figure 1. Representative images are shown. Scale bar = 100 μM. (**B**) Proliferation and differentiation of fusion-competent myoblasts were quantified as in Figure 1 (n=4). Significant differences were identified by Student's t-tests (*p<0.004 for human cells; *p<2×10^−20^ for mouse cells). (**C**) Immunoblotting analysis of the downstream effector pErk1/2 in the MAPK signaling pathway in human and mouse myoblasts serum starved for one hour then treated for 20 minutes with 50% differentiation medium and 50% of the specified medium +/− a MEKi (10 μM). (**D**) Quantification of pERK1/2 expression in human myoblasts. The relative expression level was normalized by GAPDH and presented as the expression level relative to that of human myoblasts treated with just differentiation medium. Significant differences were identified by Student's t tests (*p<0.02 and **p<0.05). Error bars indicate standard error of the mean (n=4).

hESC-conditioned medium loses its rejuvenating effect on mouse stem and progenitor cells when the MAPK pathway is inhibited [14]. Considering the evolutionary conservation of the pro-myogenic activity of hESC-produced proteins between mouse and human, we examined the dependence of hESC-produced proteins on MAPK in human muscle progenitor cells. With only 2% horse serum, differentiation medium is low in mitogens and growth factors, and quickly (in 48-72 hours) promotes the differentiation of myoblasts into multinucleated myotubes. MAPK inhibition, via incubation of human muscle progenitor cells for 72 hours in a 50/50 mixture of differentiation medium and hESC-conditioned Opti-MEM along with a mitogen-activated protein kinase-kinase inhibitor (MEKi), drastically inhibited the proliferation of primary human muscle progenitor cells, assayed by a BrdU incorporation pulse (Figure 2A, quantified in B), suggesting that the hESC-produced, pro-regenerative proteins signal through the MAPK pathway in human muscle progenitor cells (Figure 2A, B). These results are conserved with mouse myoblasts and with old mouse muscle regenerative cells, as confirmed by incubation of mouse cells in hESC-conditioned Opti-MEM with or without treatment with MEKi (Figure 2A, B, Supplementary Figure 2B, and as previously published [14]).

We further pursued the mechanisms by which hESC-produced proteins enhance the regenerative capacity of muscle progenitor cells, with a focus on MAPK signaling. Human and mouse muscle progenitor cells were serum starved for one hour, followed by a 20 minute culture in differentiation medium with 50% of hESC-conditioned Opti-MEM, hESC-conditioned Opti-MEM + MEKi, or Opti-MEM conditioned by differentiated hESCs as a negative control. Immuno-detection by Western blotting was performed on these cells, and data were normalized to the levels of housekeeping proteins (GAPDH and cytoplasmic β-actin). The levels of detected phosphorylated-activated proteins (for example, pERK1/2) were also normalized by the levels of their respective total protein (for example total ERK1/2). As shown in Figure 2C (quantified in D), hESC-produced factors induced higher levels of pERK1 and pERK2 in human and mouse muscle progenitor cells, as compared to cells cultured in medium conditioned by differentiated progeny of hESCs. As anticipated, MEKi treatment down-regulated pERK1 and pERK2. These data suggest that proteins produced by hESCs, but not by their differentiated progeny, exert a cross-species, conserved activation on the MAPK pathway in myogenic cells.

**Figure 3:**
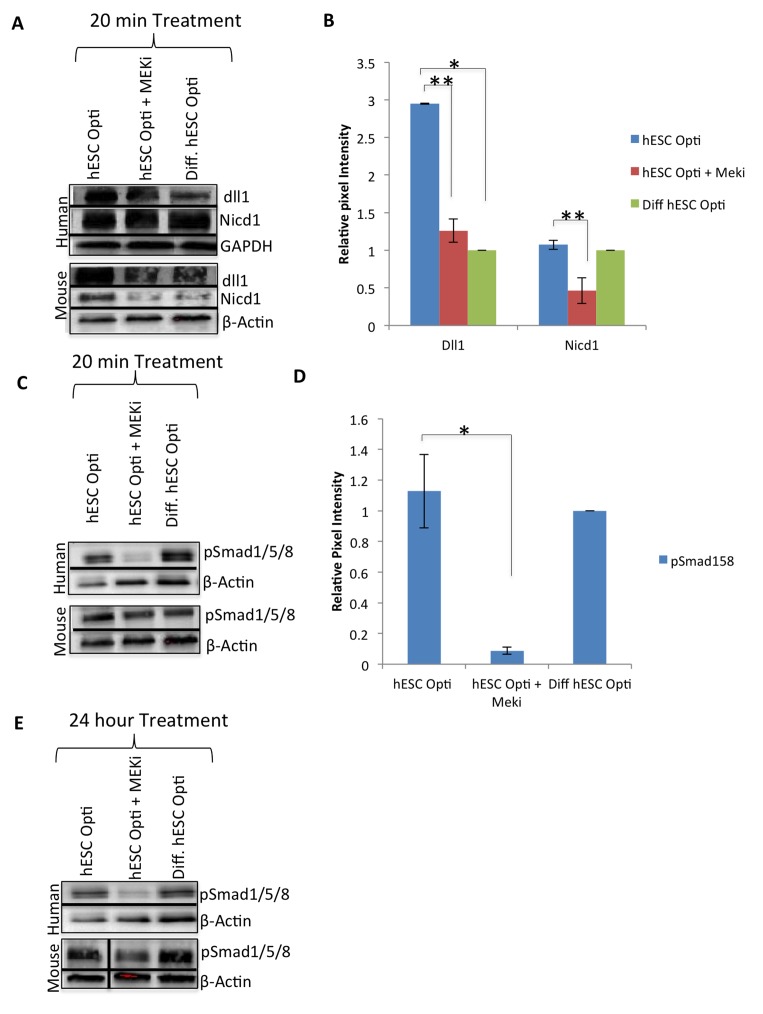
hESC-produced factors act on multiple biochemical pathways Western immunoblotting analysis with GAPDH or β-actin as loading controls, from human and mouse myoblasts serum starved for one hour then treated for 20 minutes with 50% differentiation medium and 50% specified medium, with or without a MEKi (10 μM) to analyze crosstalk with MAPK pathway. (**A**) Immunoblot of the Notch pathway, specifically Delta-1 (dll1) and active Notch-1 (Nicd1), (**B**) Quantification of Dll1 and Nicd1 expression in human myoblasts. The relative expression level was normalized by GAPDH and presented as the expression level relative to that of human myoblasts treated with just differentiation medium. Significant differences were identified by Student's t tests (*p<0.002 and **p<0.05). Error bars indicate standard error of the mean (n=3). (**C**) Western immunobloting analysis of BMP pathway proteins pSmad 1/5/8 with β-actin as a loading control, as in (**A**). (**D**) Quantification of pSmad1/5/8 expression in human myoblasts. The relative expression level was normalized by βactin and presented as the expression level relative to that of human myoblasts treated with just differentiation medium. Significant differences were identified by Student's t tests (*p<0.05). Error bars indicate standard error of the mean (n=3). (**E**) Western immunobloting analysis of BMP pathway protein pSmad 1/5/8 with βactin as a loading control, from human and mouse myoblasts treated for 24 hours with 50% differentiation medium and 50% specified medium +/− MEKi (10 μM).

Several signaling pathways (such as TGF-β/pSmad2,3, BMP/pSmad1,5,8, canonical Wnt and Delta/Notch), have been shown to be crucial for muscle regenerative capacity and to be deregulated with age [2]. Western blotting analysis as above showed no significant up or down-regulation of TGF-β/pSmad2,3, BMP/pSmad1,5,8, and canonical Wnt/β-catenin signaling in primary human and mouse muscle progenitors upon exposure to the hESC-produced factors after 20 minutes or even after 24 hours (Supplementary Figure 3A, B). In contrast, the Notch ligand Delta-1 and truncated/activated Notch-1 (NICD1) were significantly up-regulated by exposure to the hESC-produced proteins, as compared to the differentiated progeny of these hESCs that lack pro-regenerative activity (Figure 3A, quantified in B). Similar to MAPK/pERK, the induction of Delta/Notch was conserved between human and mouse primary muscle progenitors, although in mouse progenitors there was a more apparent upregulation of NICD1. As compared to mouse cells, basal Notch signaling remained relatively high in human muscle progenitors transferred from highly mitogenic growth medium into the mitogen-low differentiation medium or Opti-MEM, even after serum starvation (Supplementary Figure 3C), suggesting that Notch-1 that has been activated in growth medium persists in human muscle progenitors (Figure 3A, B).

Considering the prominent evolutionary-conserved cross talk between MAPK and Notch pathways, we examined whether MAPK/pERK signaling is required for the activation of the Delta/Notch pathway by the hESC-produced factors [3, 6]. Western blotting was performed on human and mouse muscle progenitors that were serum starved for one hour and then exposed for 20 minutes to hESC-produced proteins in the absence or presence of MEKi. As shown in Figure 3A and B, Delta-1 was up-regulated by the hESC-conditioned medium in both human and mouse primary muscle progenitor cells, and NICD upregulated in mouse cells, but not when MAPK signaling was inhibited. Expression levels of Wnt/β-catenin and TGF-β/pSmad2,3 pathway components, on the other hand, were not regulated by hESC-produced proteins, or affected by MEKi, after a 20 minute or even 24 hour exposure (Supplementary Figure 3A, B). Finally, even though BMP/pSmad1,5,8 signaling was not activated by the hESC-conditioned medium in human or mouse primary muscle progenitors (Figure 3C, D), this pathway was dependent on intact MAPK signaling in myoblasts of both species: phosphorylation of pSmad1,5,8 was significantly down-regulated by the MEK inhibitor by 20 minutes of treatment in human and mouse cells (Figure 3C, D), and remained inhibited even after 24 hours of treatment in human myoblasts (Figure 3C, D and E).

These results suggest that hESC-produced proteins enhance the regenerative capacity of postnatal mouse and human muscle progenitors by quickly activating MAPK/pERK and Delta/Notch signaling, and are suggestive of crosstalk between MAPK and Notch signaling pathways.

### Down-stream effectors of hESC-produced factors in primary myogenic human cells

**Figure 4:**
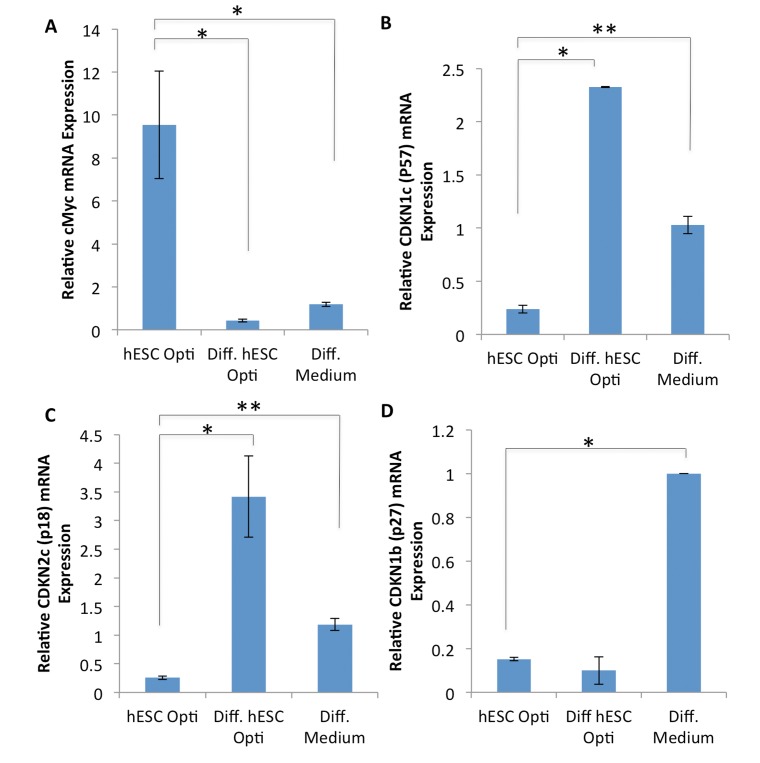
Expression analysis of MAPK regulated genes (**A**-**D**) Quantification of downstream CDK inhibitor expression genes by qRT-PCR in human myoblasts treated for 72 hours with 50% differentiation medium and 50% of the medium specified, with daily medium changes. The relative expression level was normalized by GAPDH. Significant differences were identified by Student's t tests (*p<0.05 for c-Myc, *p<7×10^−6^ and **p<0.005 for p57,*p<0.05 and **p<0.01 for p18, *p<3×10^−6^ for p27). Error bars indicate standard error of the mean (n=2-4).

To further understand the mechanisms by which hESC-produced factors enhance the regenerative responses of muscle progenitor cells, gene expression analysis was performed on the downstream targets/effectors of the above signaling pathways, with a specific focus on the regulators of cell cycle progression. Human myoblasts were cultured for 72 hours in a 50/50 mixture of differentiation medium plus medium conditioned by either hESCs or differentiated hESCs, or in 100% of differentiation medium, followed by mRNA expression analysis by RT-PCR. As shown in Figure 4A-C, hESC-produced factors induced expression of c-Myc and down-regulated the levels of p57 and p18 cyclin dependent kinase (CDK) inhibitors. Interestingly, the level of another CDK inhibitor p27 was down-regulated by both pro-myogenic medium conditioned by hESCs and control medium conditioned by their differentiated progeny (Figure 4D). However, the expression of an additional regulator of cell cycle progression, cdc25a, was not influenced by either hESC or differentiated hESC-conditioned medium (data not shown). These results demonstrate that hESC-produced proteins do not down-regulate cell cycle inhibitors broadly, but rather influence a specific subset of the downstream effectors of cell proliferation in human muscle progenitor cells. Con-sidering that these downstream effectors are direct targets of the MAPK/pERK and Delta/Notch pathways, such signaling cascades could interact in a combinatorial fashion to induce c-Myc and repress p18 and p57 in muscle progenitors, promoting their regenerative capacity.

### Proteomic analysis of hESC-conditioned Opti-MEM

Considering that medium conditioned by hESCs, but not their differentiated progeny, has pro-regenerative activity (Figure 1), which is lost upon Proteinase K treatment [14, 17], we performed a comparative proteomics antibody array analysis of these two media (Materials and Methods). Replicate experiments indicated specific proteins that were more abundant in the hESC-conditioned medium compared with medium conditioned by differentiated hESCs, and the top candidates are listed in Table 1. Consistently abundant were factors that signal through the MAPK, TGF-β, Interleukin, and apoptosis pathways (Table 1). Given that heparin-binding hESC-produced factors are responsible for the pro-myogenic and regenerative properties, that FGFs bind heparin [16], and that MAPK signaling is crucial for the regenerative effects of hESC-conditioned medium, from among the growth factors and cytokines that were highly up-regulated in the hESC-conditioned medium, we first focused on the FGFs.

**Table 1:** List of most highly enriched proteins in hESC-conditioned medium

Ranking	Protein name	Function/pathway
1	FGF Basic	Growth factor/MapK
2	FGF-19	Growth Factor/MapK
3	Angiogenin	Ribonuclease, activates ERK
4	BTC	Betacellulin growth factor
5	IL-13 R alpha 2	Cytokine receptor
6	Siglec-5/CD170	Sialic acid IgG like lectin, recruits phosphatases to attenuate signaling
7	IL-15	Cytokine
8	APJ	Apelin receptor, GCPR signals motility
9	IGFBP-2	Insulin like Growth Factor Binding Protein/MapK
10	Chordin-Like 1	TGF-β pathway antagonist
11	GASP-1 / WFIKKNRP	TGF-inhibitor, protease inhibitor, binds myostatin
12	MFRP	Membrane Frizzled related protein, Wnt pathway
13	IL-10 R alpha	Cytokine receptor
14	Chem R23	Chimerin receptor 23, chemokine
15	HB-EGF	Growth factor/MapK
16	FGF-6	Growth factor/MapK
17	HGF	Hepatocyte Growth Factor-Scatter Factor/MapK
18	IL-16	Cytokine
19	IL-7 R alpha	Cytokine receptor
20	TRAIL R3 / TNFRSF10C	TNF receptor family, antagonist to apoptosis
21	BMP-6	TGF-β ligand
22	IL-1 F9 / IL-1 H1	Cytokine
23	IL-1 β	Cytokine
24	Kremen-2	Kringle containing membrane protein 2, antagonist to Wnt
25	TRAIL R4 / TNFRSF10D	Antagonist to apoptosis
26	CXCR1 / IL-8 RA	Cytokine receptor
27	Ck β 8-1 / CCL23	Cytokine
28	β-Catenin	Wnt pathway
29	FGF-13 1B	Growth factor/MapK
30	TRAIL / TNFSF10	Modulates apoptosis
31	CCL14 / HCC-1 / HCC-3	Cytokine
32	FGF-4	Growth factor/MapK

### hESC candidate proteins FGF 2, 6, and 19 enhance myogenesis and old muscle regeneration

We tested the myogenic effects of fibroblast growth factors found to be enriched in hESC-conditioned medium, specifically FGF2, 6, and 19, first singly on mouse myoblasts in a dose-dependent manner, and then in combinations. We assayed cell proliferation (BrdU incorporation) and myogenic differentiation (eMyHC+) of myoblasts conditioned for 24 hours in low-mitogen differentiation medium supplemented with recombinant FGF 2, 6, or 19. FGF2 enhanced mouse myoblast proliferation in a dose-dependent manner, and FGF6 also enhanced proliferation but to a lesser degree. FGF19 alone, however, had minimal effects on proliferation (Supplementary Figure 4A). Furthermore, both FGF2 and 6 inhibited differentiation, also in a dose-dependent manner (Supplementary Figure 4B). FGF19, on the other hand, did not inhibit differentiation (Supplementary Figure 4B). It is important to note that human FGF19 does not exist in mouse cells, and its closest homologue is FGF15 [18]. Curiously, when added in combination, FGF6 and 19 were pro-proliferative and inhibited differentiation to a degree similar to that of FGF2 alone (Supplementary Figure 4C, D). Based on the dosage assays, FGF2-induced proliferation was optimal at 30 ng/mL; and the FGF6 and 19-induced proliferation plateaued at 30 ng/mL (Supplementary Figure 4).

**Figure 5:**
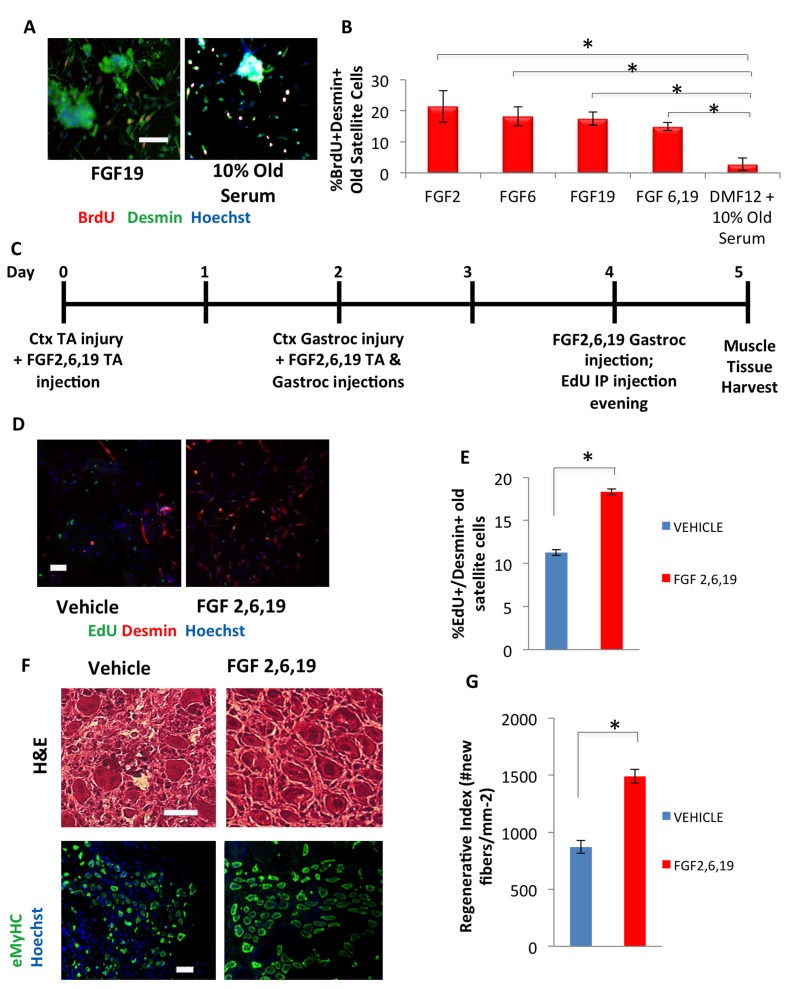
hESC candidate factors FGF2, 6, and 19 exhibit a pro-myogenic effect in an old environment and enhance old muscle regeneration *in vivo* (**A**) Old injury activated myofiber-associated satellite cells were isolated from 3 days post cardiotoxin-induced muscle injury, and cultured overnight in DMEM/F12 with 10% of old serum and 10 ng/mL FGF 2, 6, 19, or FGFs 6 and 19 in combination, followed by a 2 hour BrdU pulse to label proliferating cells before fixation. Immunofluorescence was performed with Desmin (green) and BrdU (red), with Hoechst (blue) labeling all cell nuclei. Representative images are shown. Scale bar = 100 μM (**B**) Proliferating myogenic Desmin+/BrdU+ satellite cells were counted. Results are displayed as the mean percent of BrdU+/Desmin+ proliferating satellite cell cells. Error bars indicate standard error of the mean (n=4). Significant differences were identified by Student's t tests (*p<0.0004). (**C**) Schematic of *in vivo* FGF2, 6, and 19 injection. EdU was injected (intraperitoneal) at Day 4 to label proliferating, fusion-competent myoblasts. (**D**) Old injury activated myofiber-associated satellite cells from at 3 days post cardiotoxin-induced muscle injury (Day 5 in schematic), and cultured overnight in Opti-MEM with 10% of their respective old serum, followed by cell fixation. Immunofluorescence was performed with Desmin (red) and EdU (green), with Hoechst (blue) labeling all cell nuclei. Representative images are shown and demonstrate that FGFs have a pro-myogenic and rejuvenating effect. Scale bar = 100 μM (**E**) Proliferating myogenic Desmin+/EdU+ satellite cells were quantified by cell scoring as in Figure 1. Results are displayed as the mean percent of EdU+/Desmin+ proliferating satellite cell cells +/−SD (n=4). Significant differences were identified by Student's t tests (*p<0.03). (**F**) Cryosections (10 μm) of old Tibialis Anterior muscles were analyzed by hematoxylin/eosin (H&E) staining and immunostaining for embryonic myosin heavy chain (eMyHC, shown in green). Hoechst stains nuclei (blue). Scale bars = 100 μM (**G**) Regeneration of old mice Tibialis Anterior muscle 5 days post injury, that received candidate factors FGF 2,6, and 19 or vehicle, were quantified from muscle sections and are presented as Regenerative Index, the number of newly regenerated myofibers per square millimeter of injury site. Error bars indicate standard error of the mean (n=4). Significant differences were identified by Student's t tests (*p<0.0007).

We next tested the effects of these embryonic candidate factors on human myoblasts to determine whether their activities were evolutionarily conserved. FGF2, 6, and 19 all induced proliferation of human myoblasts in a dose-dependent manner (Supplementary Figure 5A, C). However, similar to mouse cells, FGF19 did not inhibit differentiation, while FGFs 2 and 6 did (Supplementary Figure 5B, C). When tested on freshly isolated, injury-activated old muscle regenerative cells, each exogenous FGF individually enhanced and rejuvenated myogenesis, based on the numbers of newly generated BrdU+/Desmin+ proliferating muscle progenitors that migrate off freshly isolated regenerating old muscle fibers in culture (Figure 5A, B). Additionally, while FGF19 had very mild effects on primary cultured mouse myoblasts, this growth factor displayed a potent pro-regenerative activity on freshly isolated muscle stem cells (Figure 5A, B). These results suggest that either FGF19 signaling is different between the muscle repair cells and their more differentiated, cultured progeny, or that a milder activation of cell proliferation but without inhibition of differentiation, is still effective for the rejuvenation of the myogenic capacity of old satellite cells.

When tested *in vivo*, a mixture of FGF2, 6, and 19 significantly improved the activation of the aged muscle stem cells following injury and rejuvenated the repair of old injured skeletal muscle, based on the numbers of newly-generated proliferating muscle progenitors at 3 days post injury and the robust formation of the de-novo eMyHC+ muscle fibers at 5 days after the injury (Figure 5C-G).

**Figure 6:**
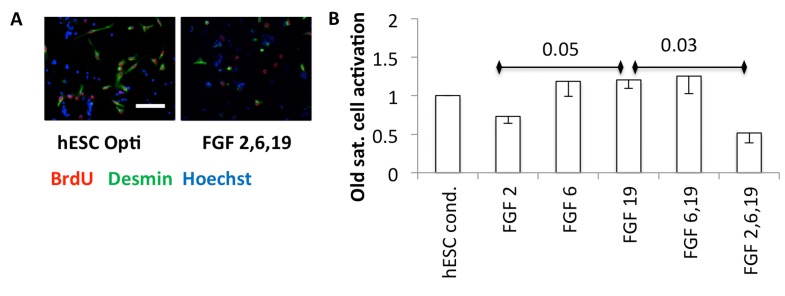
(**A**) Quiescent satellite cells were isolated from uninjured muscle and plated into quiescent medium (DMEM with 1% horse serum final concentration [43]), with an equal volume of Opti-MEM conditioned by hESCs or Opti-MEM with the indicated FGFs at 30 ng/ml each, cultured for one day and then BrdU added overnight before fixation and immunoanalysis. Representative images are shown. Scale bar = 100 μM (**B**) Quantification. The activated satellite cells are the mean fraction of myogenic desmin +ve cells that are proliferating (BrdU+ve), normalized to the hESC condition, from three experiments, with − 1 SEM indicated (n=3). P-values are shown between FGF2 and FGF19, and FGF19 and FGF 2,6,19.

Growth factors are known to enhance proliferation. However, the activation of satellite cells requires both breakage of quiescence and mitotic promotion, and FGF2 alone has been shown to be insufficient to break quiescence [17, 19, 20]. To examine any possible effects of hESC secreted pro-regenerative factors on activation, quiescent satellite cells were isolated from uninjured mouse muscle and plated in a low-mitogen plating medium, with an equal volume of hESC conditioned Opti-MEM or FGFs added in Opti-MEM. Typically in vivo and in vitro, satellite cells break quiescence and enter S-phase within 30 hours [21] (and our unpublished observations). BrdU was added after plating quiescent cells, and 36 hours later the cells were fixed for immunoanalysis for myogenicity (desmin) and progression through S-phase of cell cycle (BrdU). In hESC-conditioned medium, some myogenic satellite cells broke quiescence, and the fraction of cells that were activated was relatively high (Figure 6A, B). Interestingly, FGF6 and FGF19 also encouraged cells to break quiescence, but FGF2 failed to activate satellite cells and even appeared to interfere with this effect of FGF6 and FGF19 (Figure 6A, B).

These results demonstrate that FGFs 2, 6, and 19 are not only enriched in serum-free medium conditioned by the hESCs, but are sufficient for pro-myogenic effects in mouse and human systems and additionally in their combination, to enhance the repair of old injured muscle.

## DISCUSSION

Our initial study demonstrated that embryonic stem cells produce soluble proteins that robustly enhance adult muscle stem cell function even in an aged environment, and that production of such proteins is lost when these cells differentiate [14, 17]. Furthermore, the MAPK pathway was determined to be critical in modulating the activity of these embryonic protein(s) [14]. These findings are supported by microarray analysis conducted on cardiomyocytes subjected to hESC-conditioned medium, demonstrating that MAPK pathway signaling was among the main induced signaling cascades [22]. Here we uncover the molecular identity of active hESC-produced proteins and demonstrate that specific FGFs are sufficient to enhance mouse and human myogenesis.

While FGFs had significant effects on cell proliferation of human and mouse myogenic progenitors, antibody neutralization of FGF-2, FGF-6 or FGF-19 did not significantly reduce the pro-myogenic properties of hESC conditioned medium (not shown), suggesting that as expected and detected by proteomic antibody array (Table 1), many other active growth factors and MAPK ligands are secreted by the hESCs. Ultimately, the precise molecular definition of most of the pro-regenerative proteins from the hESC secretome will allow one to design optimal therapeutic applications with low off-target and side effects.

Old muscle stem and progenitor cells fail to break quiescence and proliferate in response to muscle injury largely due to a decrease in physiologic Notch signaling, and when down-stream Notch effectors become lacking, muscle stem cell prematurely differentiate into myofibers, both of which interferes with cellular homeostasis and results in scar tissue formation rather than newly formed muscle fibers [6]. Therefore, the pro-proliferative activities of the heparin-binding, hESC-secreted factors FGF2, 6, and 19 have clear therapeutic potential for enhancing old muscle progenitor cell function during muscle injury and repair. While FGF2 and 6 were able to promote proliferation and inhibit differentiation of human muscle progenitors, FGF19 mildly induced proliferation. FGF2 is a well-known mitogen and critical component of adult and embryonic stem cell culture medium [23], and enhances the proliferation of already activated myoblasts [17, 24, 25]. It is also known to promote angiogenesis, osteogenesis, wound healing, and tumorigenesis [26, 27] and to become deregulated in muscle with age [28]. FGF6, interestingly, is restricted almost exclusively in expression to the myogenic lineage during embryonic development, and at high doses FGF6 activates proliferation of muscle progenitor cells, while at low doses FGF-6 directs cell differentiation [29]. The role of FGF-6 in adult myogenesis is much less known, but ectopic expression of both FGF-2 and FGF-6 in transplanted myoblasts increased their contribution to de-novo muscle formation [29, 30]. FGF19, unlike the auto-paracrine FGF2 and 6, has both local and systemic endocrine effects [18]. FGF19 acts as a hormone to regulate nutrient metabolism, it is expressed in both neuroectoderm and mesoderm during embryonic development, and is known to induce ear formation [31, 32]. However, a role for FGF19 in muscle regulation has not been characterized prior to our work. While FGF2 is known primarily to bind FGFR1, FGF6 binds FGFR1 and 4, and FGF19 binds FGFR4, and these ligands can also induce signaling through other somewhat promiscuous FGF receptors [27, 32, 33]. Overall, the FGFs thus have complex, multifunctional roles in development and tissue regeneration, but we just started to uncover their influence on the regenerative capacity of adult and old tissue stem cells. It will be interesting to find out how differences in downstream effectors allow FGF2 and 6 but not FGF19 to inhibit differentiation and to understand in molecular terms, how FGF19 exerts its robust positive activity on the myogenicity of aged muscle stem cells. While FGF2, 6, and 19 were among the most highly upregulated by hESCs proteins, it is important to note that hESC-produced factors enhanced proliferation and inhibited differentiation to a greater extent than FGFs added alone or in combination, suggesting that there may be other factors in the conditioned medium that contribute to the observed rejuvenating effect. Deciphering the molecular identity of these boosters of tissue repair is our future interesting goal.

While the emerging paradigm is that FGF2 cannot promote the breakage of quiescence of muscle stem cells [17, 19, 20], FGF6 and 19 appear to be potent inducers of resting satellite cells to enter the cell cycle. Since miR-489 has been previously shown to be important for the breakage of satellite cell quiescence [34], it would be interesting to study whether FGF6 and FGF19 induce miR-489 or synergize with its effects. Importantly, FGF2 appeared to counteract the positive effects of FGF6 and FGF19 on the activation of quiescent satellite cells, suggesting an antagonism between these ligands, which may manifest through cross-talk between receptors, MAPK, AKT/mTOR, or other interactive signaling pathways that are known to be induced by the FGFs [33, 35].

Further analysis revealed that gene expression, not of the traditional CDK inhibitors, but a very specific sub-set of cell cycle regulators – p18, p57 and c-Myc – became altered and are thus likely responsible for transducing the pro-regenerative activity of the hESC produced proteins. In agreement with our findings, these molecules have been demonstrated to act downstream of the MAPK, Notch, and TGF-β/BMP signaling pathways in other cell types [36, 37]. c-Myc is a well-known target of MAPK signaling and inducer of proliferation [38, 39]. p18, an INK4 family member, suppresses CDK4 or CDK6 during the G1 stage of the cell cycle, and inhibits the self-renewal of adult stem cells [40]. Therefore, hESC factor-induced down-regulation of p18 in adult human myogenic progenitors may enhance their function by enhancing self-renewal. p57, a KIP2 family member, also inhibits proliferation by suppressing several G1 cyclin/CDK complexes [41]. One characteristic of aged muscle is the up-regulation of various CDK inhibitors by increased TGF-β signaling with concurrent lack of Notch-1 activation, leading to the failure of satellite cells to activate and proliferate after muscle injury [42]. hESC-secreted, heparin-binding factors can counteract this effect to enhance old muscle regeneration [17], and here we now show this is mediated through attenuation of specific CDK inhibitors via a key evolutionary and developmentally conserved signaling cross-talk between MAPK and Notch.

One important implication of our work is that identifying and reconstructing a “youthful” stem cell niche may have long-term beneficial effects on tissue regeneration, in contrast to drastic modulation of a single cell-fate regulatory pathway, that could have deleterious side effects by promoting oncogenic transformation and/or inducing cellular senescence [14]. In this regard, the composition and concentrations of the defined human FGFs that were determined and investigated here may be translatable to the clinic and yield novel strategies for the enhancement of tissue repair in the elderly, ultimately leading to a biochemically defined cocktail of physiological molecules that is able to delay and even reverse the onset of human aging.

## EXPERIMENTAL PROCEDURES

### Animals

Young (2-3 month old) and old (22-24 month old) C57BL6/J mice were purchased from the Jackson Laboratory and the NIH. The animal experimental procedures were performed in accordance with the Guide for Care and Use of Laboratory Animals of the National Institutes of Health, and approved by the Office of Laboratory Animal Care, UC Berkeley.

### Muscle fibers and muscle stem cell isolation

Injured TA muscle was dissected from healthy young and old mice and processed to myofibers with associated muscle satellite cells by collagenase digestion as published [43, 44]. To obtain satellite/myofiber associated regenerative cells, myofibers were further digested Dispase and Collagenase as published [44].

### Cell Culture

Adult human myoblasts were from a young adult [14], cultured from passage 10 to 14 in Ham's F-10 (Gibco), 10% Bovine Growth Serum (Hyclone), 30 ng/mL FGF2, and 1% penicillin–streptomycin on Matrigel (BD Biosciences) coated plates (1:100 Matrigel:PBS), at 37C and 5% CO2. For experimental conditions involving immunostaining, human cells were plated at 10,000 cells/well in Matrigel coated 8-well chamber slides (1:100 Matrigel: PBS), and cultured for 72 hours with daily re-feedings at 37C in 10% CO2 incubator prior to fixation with 70% ethanol at 4°C. Mouse myoblasts were cultured and expanded in mouse growth medium: Ham's F-10 (Gibco), 20% Bovine Growth Serum (Hyclone), 5 ng/mL FGF2 and 1% penicillin–streptomycin on Matrigel coated plates (1:300 Matrigel: PBS), at 37°C and 5% CO2. For experimental conditions involving immunostaining, mouse cells were plated at 40,000 cells/well on Matrigel coated 8-well chamber slides (1:100 matrigel: PBS) and cultured for 24 hours at 37°C in 10% CO2 incubator prior to fixation with 70% ethanol at 4°C. All experiments using a MEK inhibitor were treated with 10 μM MEK1/2 Inhibitor (U0126, Cell Signaling Technologies).

Human embryonic stem cells (H9 and H7 lines), were cultured in mTeSR-1 (Stem Cell Technologies) as published [17], and the heparin binding fraction of hESC-Conditioned Medium was prepared as published [17].

### Cell-Starvation–Mechanism Analysis

Human myoblasts were plated at 200,000 cells/well in a 6-well plate. Mouse myoblasts were plated at 300,000 cells/well in a 6-well plate. Plate wells were coated at 1:100 ECM: PBS. Cells were allowed to stick in human growth medium and mouse growth medium, respectively, for 1 hour at 5% CO_2_ at 37°C prior to 2 washes in serum-free medium (DMEM (Gibco) + 1% sodium pyruvate + 1% penicillin streptomycin). Cells were then treated 10 seconds in serum-free medium with 10μg/mL protamine sulfate for 10 seconds in order to remove any remaining bound ligands, followed by 2 more washes in serum-free medium and a one hour incubation in serum-free medium. Cells were then treated for 20 minutes in 10% CO_2_ at 37°C in 50% Differentiation Medium (DMEM + 2%HS + 1% penicillin streptomycin) and 50% specified medium. Cells were washed with PBS and cell lysates collected and analyzed for western blot using above mentioned western blot analysis method.

### Immunocytochemistry and Quantification

Cells were fixed with 70% ethanol 30 minutes to overnight before 3, 5 minute PBS washes, 10 minute 4N HCl treatment, permeablization with PBS + 0.25% Triton X-100 for 15 minutes, 3 PBS washes, and blocking one hour in PBS + 2% FBS. The cells were then incubated with primary antibodies overnight at 4C in PBS + 2% FBS. Anti-eMyHC was from the Developmental Studies Hybridoma Bank, Anti-Desmin from Sigma-Aldrich, and rat anti-BrdU from Abcam. If stained for EdU (EdU Click-It staining kit was from Invitrogen), the HCl antibody retrieval method was not performed. Tissue sections were fixed with 70% ethanol 30 minutes to overnight before 3, 5 minute PBS washes, 2N HCl treatment for 20 minutes, permeablization with 0.25% Triton-X 100 for 30 minutes, 3 PBS washes followed by blocking 1 hour with PBS + 2% FBS. Secondary staining with fluorophore-conjugated, species-specific antibodies (Donkey anti-Rat-488, #712-485-150; Donkey anti-Mouse-488, #715-485-150; Donkey anti-Rat-Cy3 #712-165-150; or donkey anti-Mouse-Cy3 #715-165-150; all secondary antibodies from Jackson ImmunoResearch). Nuclei were visualized by Hoechst stain. For cell quantification, 25-50 20x images per replicate were taken on the Molecular Devices ImageXpress Micro automated epifluorescence imager, followed by automated cell quantification using the multiwavelength cell scoring module within the MetaXpress analysis software.

### Western blotting

Human or mouse primary muscle progenitor cells were lysed in RIPA buffer (50 mM Tris, 150 mM NaCl, 1% NP40, 0.25% sodium deoxycholate and 1 mM EDTA, pH 7.4) containing 1X protease inhibitor (Roche), 1 mM Phenylmethylsulfonyl fluoride (PMSF), 1 mM sodium fluoride and 1 mM sodium orthovanadate. Cell lysates were resuspended in 1X Laemmli buffer (Bio-Rad), boiled for 5 minutes and separated on precast 7.5% or 4-15% TGX gels (Biorad). Primary antibodies were diluted in 5% non-fat milk in TBS + 0.1% Tween-20, and nitrocellulose membranes were incubated with antibody mixtures overnight at 4 °C. HRP-conjugated secondary antibodies (Santa Cruz Biotech) were diluted 1:1,000 in 5% non-fat milk in TBS + 0.1% Tween-20 and incubated for 1 hour at room temperature. Blots were developed using Western Lightning ECL reagent (Perkin Elmer), and analyzed with Bio-Rad Gel Doc/Chemi Doc Imaging System and Quantity One software. Antibodies for phospho-ERK1/2, ERK1/2, pSmad1,5,8 and β-Actin, pGSK3β Y216, and Total GSK3β, were purchased from Cell Signaling. pSmad3 was purchased from Epitomics. Smad2/3, and Dll1 antibodies were from Santa Cruz Biotechnology. GapDH and Nicd1 antibodies were from Abcam, pSmad2 and β-Catenin were from Millipore.

### Proteomic analysis

Medium conditioned by hESCs or control differentiated hESCs, in duplicate, was labeled and hybridized to Raybiotech 507 antibody array slides according to the manufacturer's protocol. Briefly, conditioned Opti-MEM medium containing extracellular proteins was dialyzed against phosphate buffered saline, biotinylated, dialyzed again, hybridized to the array, washed, labeled with streptavidin-Cy3, washed, dried and the fluorescent features imaged on a Molecular Devices 4000b scanner. Data were analyzed using Genepix and Microsoft Excel software to determine relatively enriched signal in the hESC versus differentiated samples.

### RNA extraction, RT-PCR and real-time PCR

Total RNA was extracted from primary human muscle progenitor cells using Trizol reagent (Invitrogen) according to manufacturer's instructions. 1 ug of total RNA was used for cDNA synthesis with oligo dT primers (Invitrogen). For real-time PCR amplification and quantification of genes of interest, an initial amplification using specific primers to each gene of interest (realtimeprimers.com) was done with a denaturation step at 95°C for 5 min, followed by 40 cycles of denaturation at 95°C for 1 min, primer annealing at 55°C for 30 s, and primer extension at 72°C for 30 s. Real-time PCR was performed using SYBR and an ABI PRISM 7500 Sequence Detection System (Applied Biosystems). Reactions were run in triplicate in three independent experiments. The geometric mean of housekeeping gene GAPDH or β-Actin was used as an internal control to normalize the variability in expression levels and were analyzed using the 2 ^−ΔΔCT^ method described [45].

### Muscle Injury and In vivo FGF2, 6, and 19 Injection

Cardiotoxin injury was as published [46]. FGFs were administered to the site of injury at the time of injury and again one day later, 5 microliters per site, Opti-MEM (vehicle), or FGF2, 6, and 19 cocktail (100 ng/mL in Opti-MEM) at Days 0 and Day 2 for TA, and Days 2 and 4 for gastroc (See Figure 5C schematic). Mice received intraperitonial injections of EdU (50 mg/kg) at Day 4 to label proliferating cells, and muscle was harvested at Day 5.

### Tissue Immunofluorescence and Histological Analysis

Histological analysis was as published [10, 13, 47]. Cryo-sectioning was performed through the entire volume of muscle (typically 50–70 sections total, done at 200 μm intervals), thereby serially reconstituting the entire tissue, ex vivo. Regeneration and myogenic potential was quantified by examining injury sites from representative sections along the muscle (spanning the volume of injury), then by measuring the injured/regenerating area using Adobe Photoshop Elements. Myofiber regeneration was quantified by counting total newly regenerated fibers and dividing by the regeneration area.

### Quantification and Statistical Analysis

For automated quantification of immuno-fluorescent images, 25-100 20x images per replicate were taken on the Molecular Devices ImageXpress Micro automated epifluorescence imager, followed by automated cell quantification using the multiwavelength cell scoring module within the MetaXpress analysis software. Data was analyzed using Anova and P values equal or lower than 0.05 were considered statistically significant.

## SUPPLEMENTARY FIGURES


