# Supplementary material for: Mechanisms of action of hESC-secreted proteins that enhance human and mouse myogenesis
Source: Aging (Albany NY). 2014 May 11;6(8):602–20. doi: 10.18632/aging.100659 (PMC4169857; doi:10.18632/aging.100659)
Supplement: Supplementary file 1 [file aging-06-602-s001.pdf]

43. Bischoff R. Proliferation of muscle satellite cells on intact myofibers in culture. Developmental biology. 1986; 115:129-139.
44. Conboy MJ and Conboy IM. Preparation of adult muscle fiber-associated stem/precursor cells. Methods in molecular biology Clifton, NJ. 2010; 621:149-163.
45. Livak KJ and Schmittgen TD. Analysis of relative gene expression data using real-time quantitative PCR and the 2(-Delta Delta C(T)) Method. Methods. 2001; 25:402-408.
46. Conboy MJ, Karasov AO and Rando TA. High incidence of non-random template strand segregation and asymmetric fate determination in dividing stem cells and their progeny. PLoS biology. 2007; 5:e102.
47. Conboy IM, Conboy MJ, Wagers AJ, Girma ER, Weissman IL and Rando TA. Rejuvenation of aged progenitor cells by exposure to a young systemic environment. Nature. 2005; 433:760-764.

SUPPLEMENTARY FIGURES

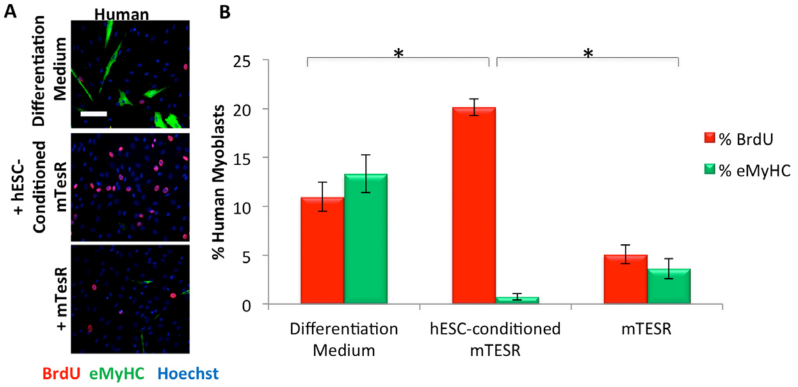

Supplementary Figure 1. hESC-Conditioned mTeSR-1, but not mTeSR-1, increases primary human myoblast Proliferation and inhibits Differentiation. (**A**) Primary human myoblasts were cultured for 72 hours in 50% differentiation medium (DMEM, 2% horse serum) plus 50% of the specified medium. A 4 hour BrdU pulse was performed before cell fixation to label proliferating cells. Immunofluorescence was performed for eMyHC (green) and BrdU (red), with Hoechst (blue) labeling all nuclei. Representative images are shown. Scale bar = 100 µM (**B**) Proliferation and differentiation of fusion-competent myoblasts were quantified by using cell profiler cell scoring software. Results are displayed as the mean percent of BrdU+ or eMyHC+ proliferating or differentiating cells +/-SEM, respectively (n=6), Significant differences were identified by Student's t tests (**p< 0.004).*

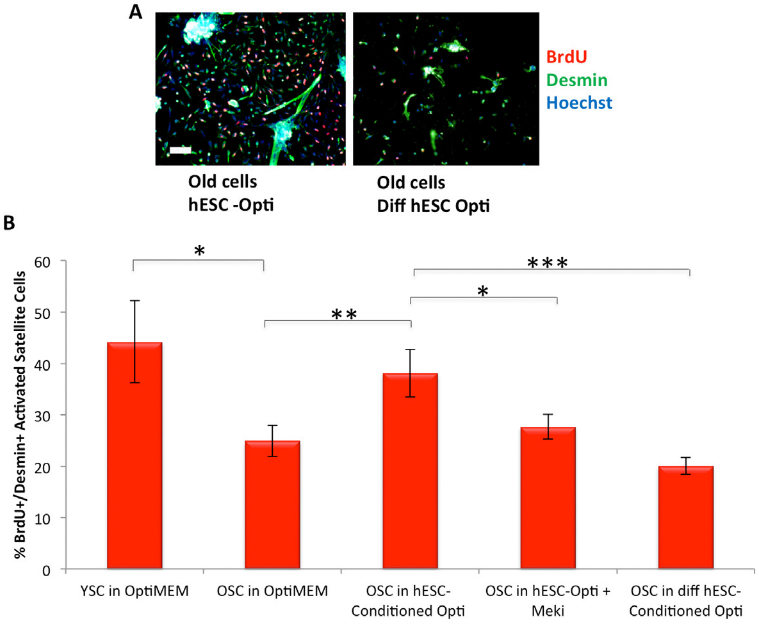

Supplementary Figure 2. hESC-conditioned medium enhanced old satellite cell activation through inducing MAPK. (**A**) Young or Old injury activated myofiber-associated satellite cells were isolated at 3 days post cardiotoxin-induced muscle injury, and cultured overnight in 50% Ham's F10 with 5% of their respective young or old serum, and 50% of the medium specified, followed by a 2 hour BrdU pulse to label proliferating cells before cell fixation. Immunofluorescence was performed with Desmin (green) and BrdU (red), with Hoechst (blue) labeling all cell nuclei. Representative images are shown. Scale bar = 100 µM (**B**) Proliferating Desmin+/BrdU+ satellite cells were quantified by cell scoring in multiple random fields of each condition. Results are displayed as the mean percent of BrdU+/Desmin+ proliferating satellite cell cells +/-SD (N=4). Significant differences were identified by Student's t tests (*$p< 0.01$, **$p<0.006$, ***$p<0.002$.)

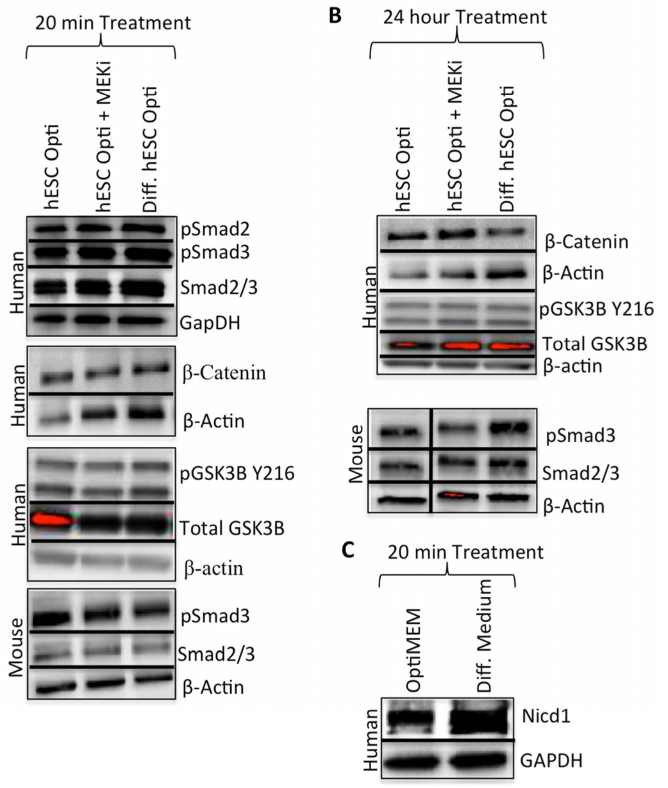

Supplementary Figure 3. hESC-produced factors Act through MapK and BMP signaling pathways, but not through TGF-β or Wnt Signaling. Western immunoblotting analysis of downstream effectors of BMP and Wnt signaling pathways in human or mouse myoblasts serum starved for one hour followed by treatment for 20 minutes (**A**) or 24 hours (**B**) with 50% Differentiation Medium and 50% Specified Medium +/- a MEKi (10 μM) to analyze crosstalk with MAPK pathway. (C) Western immunoblotting analysis of active Notch-1 in human myoblasts treated for 20 minutes with specified medium after 1 hour serum starvation.

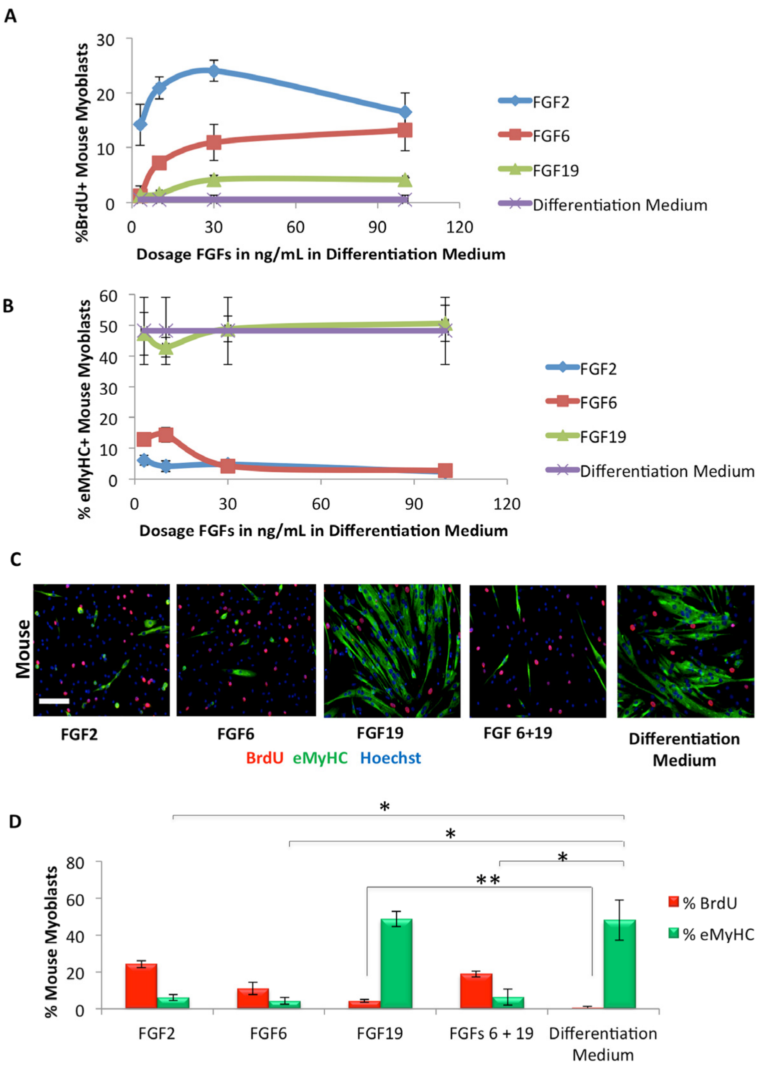

Supplementary Figure 4. hESC candidate factors FGF2,6,&19 exhibit a pro-myogenic effect on mouse myoblasts in a dose dependent manner. Primary mouse myoblasts were cultured for 24 hours in differentiation medium (DMEM, 2% horse serum) plus 0, 5, 10, 30 or 100 ng/mL FGF2, 6, or 19. A 2 hour BrdU pulse on mouse myoblasts was performed before cell fixation to label proliferating cells. Immunofluorescence was performed for eMyHC (green) and BrdU (red), with Hoechst (blue) labeling all nuclei (30 ng/ml images shown in **C**). Scale bar = 100 μM. Proliferation and differentiation of fusion-competent myoblasts were quantified as in Figure 1, and results are displayed as the mean percent of (**A**) BrdU+ or (**B**) eMyHC+ proliferating or differentiating cells +/-SD, respectively (n=6). (**D**) Quantification of FGFs added individually at the determined optimal dosage for proliferation (30 ng/mL) or in combination, as described for (A) and (B). Significant differences were identified by Student's t tests *(*p< 0.0008* and ***p<0.05*).

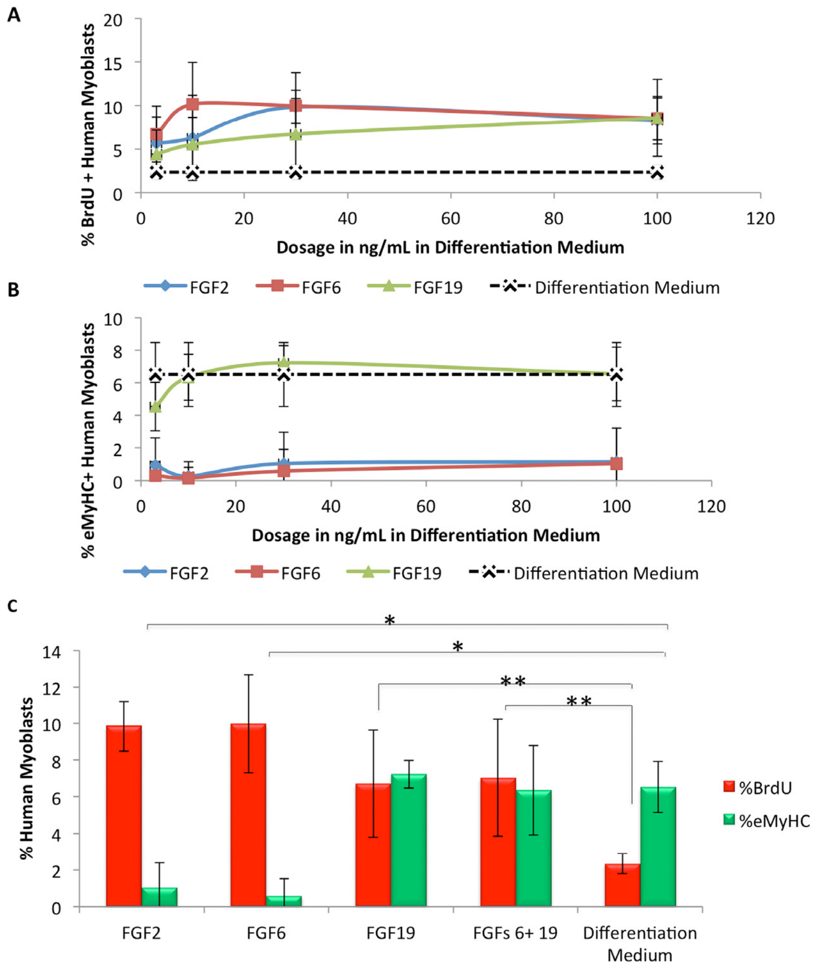

Supplementary Figure 5. hESC candidate factors FGF2, 6, 19 exhibit a pro-myogenic effect on human myoblasts in a conserved and dose dependent manner. Primary human myoblasts were cultured for 72 hours in differentiation medium (DMEM, 2% horse serum) plus 0-100 ng/mL FGF2, 6, or 19 with daily medium changes. A 4 hour BrdU pulse on human myoblasts was performed before cell fixation to label proliferating cells. Immunofluorescence was performed for eMyHC (green) and BrdU (red), with Hoechst (blue) labeling all nuclei (Images not shown). Proliferation and differentiation of fusion-competent myoblasts were quantified as in Figure 1, and results are displayed as the mean percent of (**A**) BrdU+ or (**B**) eMyHC+ proliferating or differentiating cells +/-SD, respectively (n=6). (**C**) Quantification of FGFs added individually at optimal determined dosage for proliferation (30 ng/mL) or in combination, as described for (**A**) and (**B**). Significant differences were identified by Student's t tests *(*p< 0.005* and **p<0.05*).
